# The road to successful people-centric research in rare diseases: the web-based case study of the Immunology and Congenital Disorders of Glycosylation questionnaire (ImmunoCDGQ)

**DOI:** 10.1186/s13023-022-02286-w

**Published:** 2022-03-24

**Authors:** Rita Francisco, Sandra Brasil, Carlota Pascoal, Jaak Jaeken, Merell Liddle, Paula A. Videira, Vanessa dos Reis Ferreira

**Affiliations:** 1 CDG & Allies—Professionals and Patient Associations International Network (CDG & Allies-PPAIN), Department of Life Sciences, School of Science and Technology, NOVA University Lisbon, 2819-516 Caparica, Portugal; 2 UCIBIO – Applied Molecular Biosciences Unit, Department of Life Sciences, School of Science and Technology, NOVA University Lisbon, 2819-516 Caparica, Portugal; 3 Portuguese Association for Congenital Disorders of Glycosylation (CDG), Department of Life Sciences, School of Science and Technology, NOVA University Lisbon, 2819-516 Caparica, Portugal; 4 Center for Metabolic Diseases, Department of Pediatrics, KU Leuven, 3000 Leuven, Belgium

**Keywords:** Congenital Disorders of Glycosylation (CDG), Rare diseases, People-centricity, Patient engagement, Patient empowerment, Patient recruitment, Electronic (e-) questionnaire, Social media, Web-based platforms

## Abstract

**Background:**

Congenital Disorders of Glycosylation (CDG) are a complex family of rare metabolic diseases. Robust clinical data collection faces many hurdles, preventing full CDG biological and clinical comprehension. Web-based platforms offer privileged opportunities for biomedical data gathering, and participant recruitment, particularly in rare diseases. The immunology and CDG electronic (e-) questionnaire (ImmunoCDGQ) explores this paradigm, proposing a people-centric framework to advance health research and participant empowerment.

**Objective:**

The objectives of this study were to: (1) Describe and characterize the ImmunoCDGQ development, engagement, recruitment, participation, and result dissemination strategies; (2) To critically compare this framework with published literature and making recommendations.

**Methods:**

An international, multistakeholder people-centric approach was initiated to develop and distribute the ImmunoCDGQ, a multi-lingual e-questionnaire able to collect immune-related data directly from patients and family caregivers. An adapted version was produced and distributed among the general “healthy” population (ImmunoHealthyQ), serving as the control group. Literature screening was performed to identify and analyze comparable studies.

**Results:**

The ImmunoCDGQ attained high participation and inclusion rates (94.6%, 209 out of 221). Comparatively to the control, CDG participants also showed higher and more variable questionnaire completion times as well as increased English version representativeness. Additionally, 20% of the CDG group (42 out of 209) chose not to complete the entire questionnaire in one go. Conditional logic structuring guided participant data provision and accurate data analysis assignment. Multi-channel recruitment created sustained engagement with Facebook emerging as the most followed social media outlet. Still, most included ImmunoCDGQ questionnaires (50.7%, 106 out of 209) were submitted within the first month of the project’s launch. Literature search and analysis showed that most e-questionnaire-based studies in rare diseases are author-built (56.8%, 25 out of 44), simultaneously addressing medical and health-related quality of life (HRQoL) and/or information needs (79.5%, 35 out of 44). Also, over 68% of the studies adopt multi-platform recruitment (30 out of 44) actively supported by patient organizations (52.3%, 23 out of 44).

**Conclusions:**

The ImmunoCDGQ, its methodology and the CDG Community served as models for health research, hence paving a successful and reproducible road to people-centricity in biomedical research.

**Supplementary Information:**

The online version contains supplementary material available at 10.1186/s13023-022-02286-w.

## Background

Congenital Disorders of Glycosylation (CDG) are a rapidly growing family of rare monogenic metabolic conditions. A hundred and fifty-seven genes have been linked to CDG, resulting in high intra and inter-disease clinical heterogeneity [1–3]. PMM2-CDG (MIM: 212065) was the first reported N-glycosylation defect and remains the most common CDG worldwide [4, 5]. CDG biological complexities, associated research funding limitations, disease expert and patient dispersion and scarcity hinder robust data collection. These challenges prevent full elucidation of CDG clinical picture(s) and of their natural history with immunological involvement remaining one of the least well-characterized manifestations [6].

Technological advances, namely those based on the internet have opened promising avenues not only for clinical care but also for biomedical research, particularly in the field of rare diseases (RDs). Web-based platforms have several advantages and address various gaps, namely: (i) they overcome the barrier of physical distances, hence facilitating access to and inclusion of “hard to reach” groups; (ii) they offer efficient tools for safe data collection, such as electronic (e-) questionnaires; (iii) they allow for diverse communication and empowerment strategies, and; (iv) often present as more inexpensive alternatives [7–10]. Several international initiatives have been exploring these e-health and e-research pathways. Among which stand the European Reference Networks (ERNs), whose primary aim is to serve as virtual care networks connecting medical experts and RD patients across Europe. Also, the ERNs hold great e-research potential [11, 12]. Additionally, there is the Share4Rare web-platform that congregates multi-RD research projects and the Rare Barometer e-survey program. The Rare Barometer project is led by EURORDIS and gathers worldwide data on healthcare, disease burden and other research-related topics to boost knowledge and enable policymaking in RD [13, 14].

The ERNs, Share4Rare and Rare Barometer are designed to foster people or patient-centric projects. In these programs, citizens/patients play a role beyond that of the traditional research participant. They are treated as equal partners and whose insights, preferences, values, and beliefs are continuously sought and incorporated. Importantly, people-centric projects ensure that the needs and priorities of citizens/patients are addressed [15, 16].

In biomedicine and related fields, people-centric research has been focusing on multiple health-related topics, ranging from quality of life (HRQoL) to information needs and symptom treatment prioritization. Also, the implementation of patient-fed clinical registries and surveys have reinforced the recognition of patients as unique clinical data providers [17–21]. In RDs, patients and/or family members are often obliged to become experts in their own condition since they experience the disease on a daily-basis and centralize medical information from several sources, including clinicians and other healthcare professionals. Consequently, patients and/or family members are distinctively well-positioned to provide data on health-related topics [22].

The expansion of both e-research tools and patient-centricity approaches have underlined the need to effectively educate patients in order to provide them with a skill set that supports their active and meaningful involvement in research. Hence, several training opportunities—many with a heavy internet-based component—created specifically for patients, patient representatives and family caregivers have been blossoming. Examples include the European Patients’ Academy on Therapeutic Innovations (EUPATI), the Patient-centered Outcomes Research Institute (PCORI) training courses and many other initiatives mostly driven by patient organizations (POs), such as the EURORDIS Open Academy [23–25]. Also, the need to communicate clearly with patients, facilitating access to comprehensible health information to guide their decision-making, including decisions about research participation, has been growing and gaining importance [26, 27].

The Internet again offers promising opportunities for lay people, including people living with RDs and their families, to find information, learn about and participate in research. In this context, social media networks and online patient communities have been consolidating their role as research engagement, recruitment, health information-sharing and discussion forums [8, 28–32].

This study’s rationale relies on a double-sided hypothesis: (i) that a people-centric, multi-stakeholder, multi-lingual, web-based intervention in CDG—combining research empowerment and engagement strategies—improves recruitment and participation, and; (ii) that this innovative methodology based on an e-questionnaire is an effective clinical data collection approach, namely in the field of immunology [10].

The specific aims of the present study are to:

1. Describe the people-centric methodology applied in the development of the Immunology and CDG e-questionnaire (ImmunoCDGQ);
2. Outline and characterize the ImmunoCDGQ participation, engagement, recruitment and result dissemination strategies;
3. Frame the methodology developed in the ImmunoCDGQ project by:
  3.1 Performing a literature screening and description of published studies using similar research methods (i.e., e-questionnaires adopting web-based recruitment/ dissemination strategies) in the field of RD, and;
  3.2 Doing a critical comparative analysis between published literature and the present study to make best practice suggestions on people-centric e-research approaches in health topics.

Ultimately, this work aims to guide and improve future studies employing identical methodologies.

## Methods

### Immunology and CDG electronic questionnaire (ImmunoCDGQ) people-centric methodological development, engagement, and recruitment strategies

The global dispersion of CDG families and medical experts motivated the creation of an author-built e-questionnaire tool—the ImmunoCDGQ. Description of its scientific content development, piloting, translation, and ethical submission are reported in [10] and detailed in the checklist for reporting results of internet E-Surveys (CHERRIES, Additional file 1: Table S1).

**Fig. 1:**
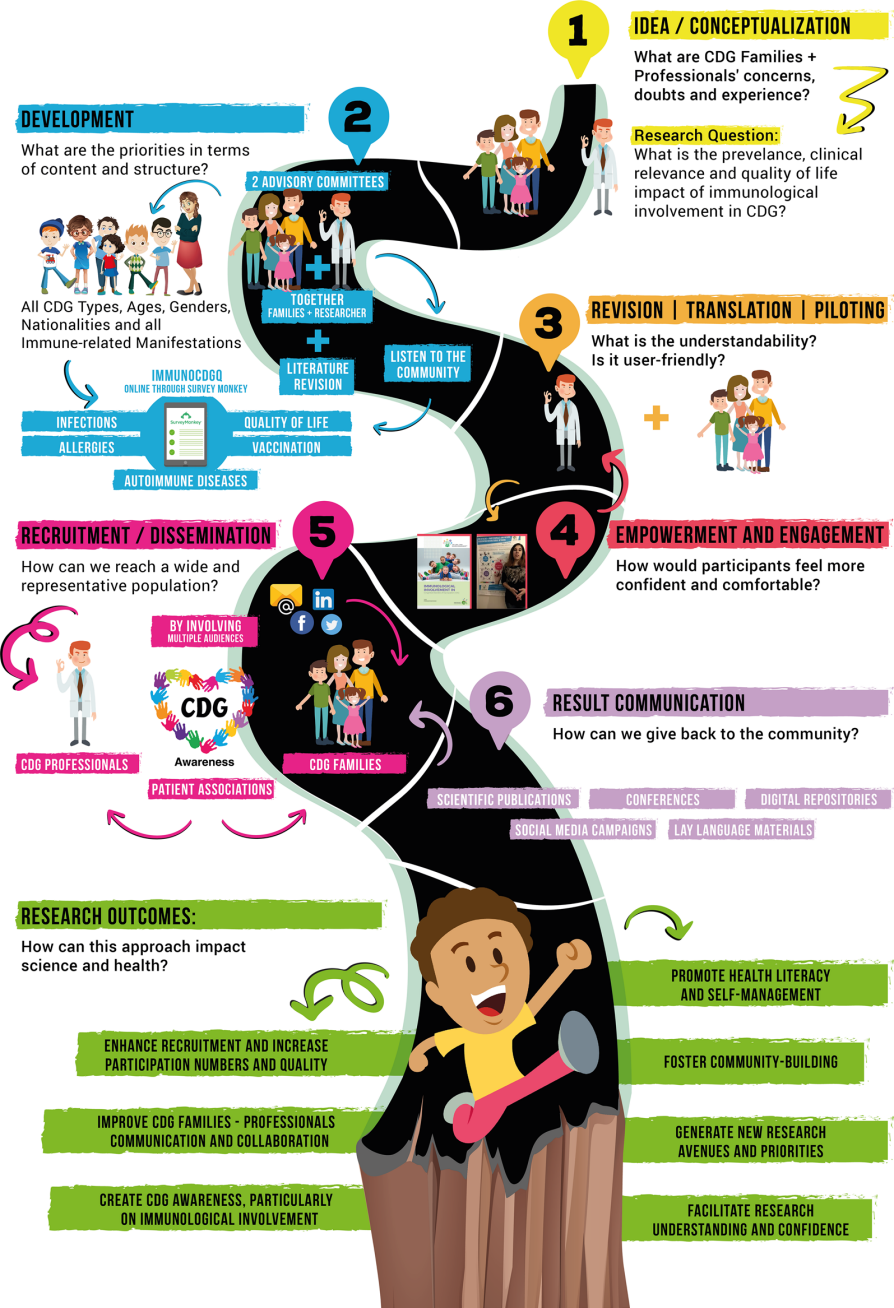
The road to people-centricity in biomedical research; Overall schematic representation of the people-centric methodology created for the development, refinement, and dissemination of the ImmunoCDGQ

The stepwise process of the ImmunoCDGQ methodological development—ranging from its ideation to the generated clinical and scientific results communication—is depicted in Fig. 1:

1. Idea/Conceptualization: Immunological involvement was selected as the research focus of the study based on: (a) repeated CDG families’ expressions of concern about immune-related manifestations, particularly infections, and; (b) the lack of solid medical and scientific information on this subject, being this knowledge gap also signaled by CDG professionals. A CDG community-needs mapping exercise resulted in the set-up and development of a patient-led international network, the CDG & Allies—Professional and Patient Associations International Network (CDG & Allies-PPAIN) which offered a collaborative platform to deploy efforts to unravel immunological involvement in CDG [16]. Accordingly, a CDG Glycoimmunology working group was integrated in the CDG & Allies-PPAIN framework [33].
2. Development: The complexity of the topic, in addition to the scarcity and dispersion of expert information led to: (a) a thorough literature review on existing medical and scientific evidence [6, 34] and; (b) the establishment of two advisory committees. One composed by 4 clinicians and 5 researchers acting as the medical/scientific board, and a CDG family advisory board formed by 5 parents. CDG patients and family caregivers were defined as the ImmunoCDGQ target audience.

This choice was made for 4 main reasons:

(i) CDG patients and family members are the stakeholder who centralize the biggest amount of information. This is particularly relevant when the medical topic is little known. Also, in this case there was prior knowledge—confirmed by the study’s advisory boards—that only a few CDG patients were followed by immunology specialists;
(ii) CDG families enabled the assessment of other to date unexplored aspects, such as immunological involvement HRQoL and information needs;
(iii) The possibility to explore health and research empowerment strategies, and;
(iv) The chance to establish a strong method to capture biomedical data directly from CDG patients and family caregivers.
  2.1 Given the exploratory nature of the study and the diversity of CDG, the ImmunoCDGQ was designed to be an inclusive tool, contemplating all CDG types and devoid of any age, gender, or geographical limits or limitations.
  2.2 To better frame and clarify the prevalence and relevance of immune-related manifestations among CDG patients and types, the ImmunoCDGQ was adapted to be administered to the general “healthy” population (ImmunoHealthyQ) which served as the control group in this study [10]. Both the ImmunoCDGQ and ImmunoHealthyQ were translated into several languages.

**Fig. 2:**
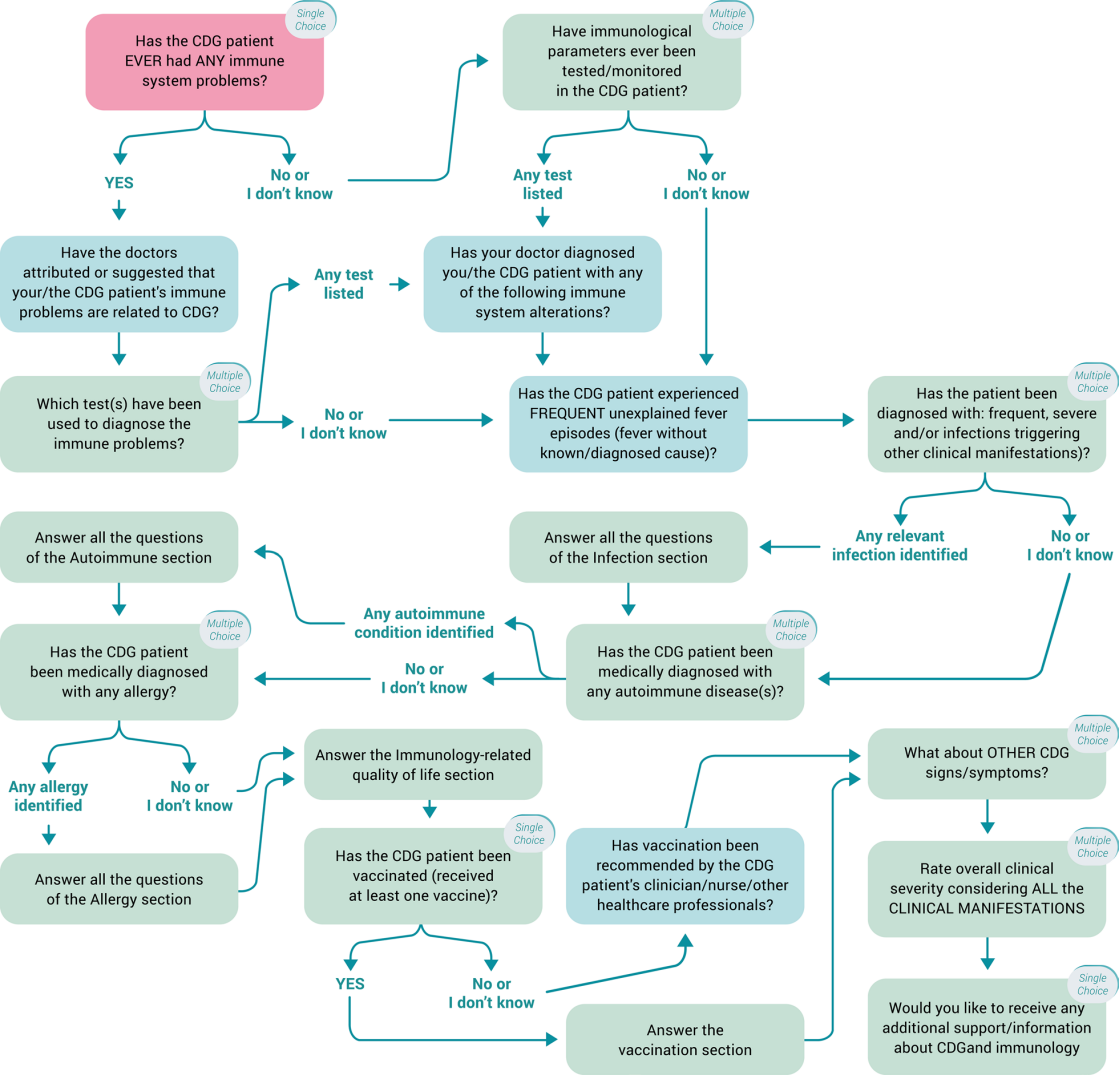
Scheme of the conditional logic design applied to the structuring of the ImmunoCDGQ; For representation simplicity, non-essential questions for the overall logical conditioning understanding have been omitted. The first question related to immunological involvement is colored in light red

Our group’s consolidated expertise in the SurveyMonkey software (Copyright #1999–2019 SurveyMonkey) weighted on the selection of this platform to develop and administer the e-questionnaires [22]. Importantly, the possibility of adding conditional logic to key questions was deeply explored and potentiated in the structural design (Fig. 2). Despite the total number of items in the ImmunoCDGQ being 58 (56 items in the ImmunoHealthyQ), at decisive questions participants were (re)directed to specific questions/sections depending on the absence/presence of immune-related signs and symptoms. This conditional logic structuring influenced the total number of mandatory items participants had to answer and it also guided data analysis (Additional file 1: Figure S1). Indeed, conditional logic facilitated the establishment and application of participants exclusion and inclusion criteria as well as enabled the creation of three categories among which participants were distributed according to their immune-related manifestations reporting: (0) without immunological involvement; (1) with immunological involvement, and; (2) with unsolved immune status (Additional file 1: Figure S1) [10].

**Fig. 3:**
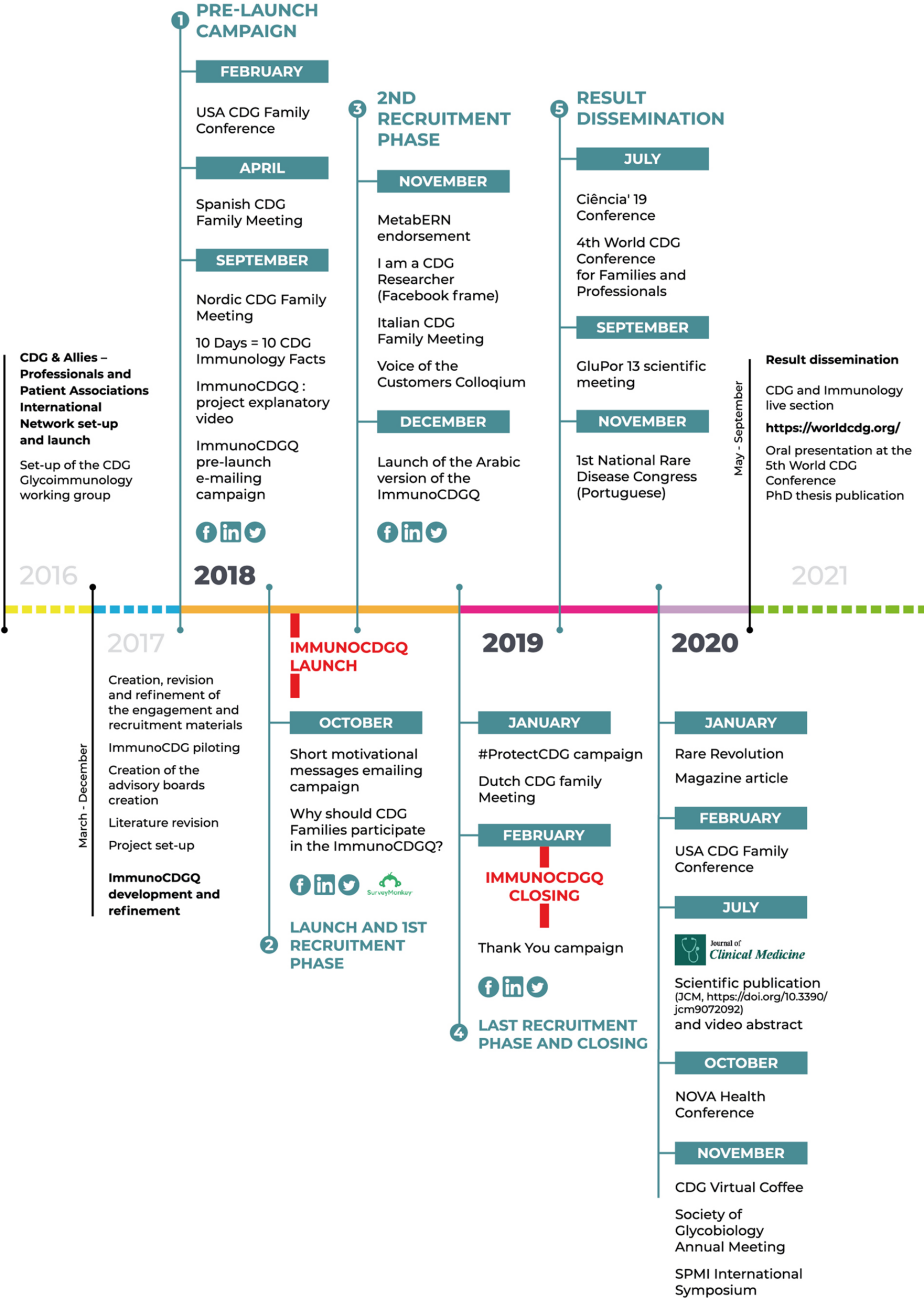
ImmunoCDGQ engagement and recruitment strategies timeline; Legend: The scheme illustrates the various explored platforms and developed resources distributed by the different communication and dissemination phases (1- Pre-launch campaign; 2—Launch and 1st recruitment phase; 3—2nd recruitment phase; 4—Last recruitment phase and questionnaire closing; 5—Result communication)

1. Revision, piloting, and translation: Besides the active involvement in the development of the questionnaire, the advisory committees reviewed its content, structure, language appropriateness and piloted it. Anticipating the global outreach of this study, questionnaire multi-lingual versions were made available. Five languages were common to both the ImmunoCDGQ and ImmunoHealthyQ, including English, Portuguese, Spanish, French and Italian. Additionally, an Arabic version of the ImmunoCDGQ was produced. Translations were performed by either (1) native speakers who concomitantly were medical/biomedical experts and sent to lay native speakers to check for understanding or (2) lay native speakers and reviewed by medical/biomedical experts for accuracy;
2. Empowerment and engagement: Once the final multi-lingual versions of the ImmunoCDGQ were obtained and prior to the official launch and active recruitment campaigns, a pre-launch campaign was set-up (Figs. 1 and 3). During this 7-month period (February 2018 to September 2018), multi-language informative materials and resources—on the project and on CDG and immunology concepts (e.g., thematic glossaries and patient-friendly guidelines)—were prepared and disseminated at CDG conferences, on social media networks and via e-mailing. These materials were centralized in the project’s webpage [35] which acted as a hub throughout the whole project (Figs. 1 and 3, Additional file 1: Tables S2 and S3);
3. Recruitment and dissemination: Following this preliminary engagement phase, active participant recruitment began with the launch of the ImmunoCDGQ on 1st October 2018 in 5 languages—English, Spanish, Portuguese, Italian and French. Participant recruitment was a highly dynamic process (Fig. 3). The evolving and adaptive nature of this phase is shown by the diversity of its associated initiatives. At the early stages of the recruitment phase, two important engagements campaigns were launched: i) the “Why should CDG Families participate in the ImmunoCDGQ?” social media campaign which was supported by many CDG families and professionals, and; ii) an e-mailing campaign based on short and motivational messages (Figs. 1 and 3, Additional file 1: Table S2, Figure S2). In addition to the already ongoing campaigns, the types of engagement strategies and partners were extended in the 2nd recruitment phase (Fig. 3). These strategies included the project endorsement by the ERN for Rare Metabolic Diseases (MetabERN), the study dissemination in two conferences, the creation of the “I am a CDG Researcher” Facebook frame campaign, and the launch of the Arabic version of the ImmunoCDGQ. Lastly, preceding the official closing date of the questionnaire (21st January 2019), a final recruitment phase occurred and encompassed the #ProtectCDG social media campaign as well as the project presentation at the Dutch CDG Family meeting. Immediately after the closing of the questionnaire, thank you posts and e-mail messages were shared (Fig. 3, Additional file 1: Figure S2). The last engagement phase was focused on:
4. Result communication: ImmunoCDGQ results were distributed and communicated at several events, platforms and using various formats targeting different audiences, i.e., scientists, clinicians, CDG patients, family members and society in general (Fig. 3, Additional file 1: Tables S2 and S3).

For a complete overview of all the engagement, recruitment and result communication materials developed throughout the entire project access Additional file 2. Social media post and email texts are included. Detailed social media metrics, languages in which the materials were made available and targeted audiences are also shown.

### Literature search and revision of e-questionnaire-based research studies in rare diseases

The Medline database PubMed and Google Scholar were selected to retrieve both indexed and grey literature. The list of keyword combinations and implemented inclusion and exclusion criteria are available in Additional file 1: Table S4.

Additional file 1: Figure S3 summarizes the search and article selection processes which were carried out by two independent researchers. Disagreements were settled through dialogue.

The initial paper screening step was based on title and abstract. The remaining articles were screened by reading the full text. Duplicates were excluded with the Mendeley duplicate removal tool.

### Statistical analysis

Statistical analysis was done using R programming language on RStudio software (version 3.6.1) and graphs created on GraphPad Prism version 7.0. Descriptive statistics were performed to analyze and report findings. Measures of central data distribution—mean (µ)—and of data variation—standard deviation (SD)—are presented and calculated using the respective R functions, mean () and sd ().

## Results

### ImmunoCDGQ attained high participation, inclusion rates and English version representativeness

**Fig. 4:**
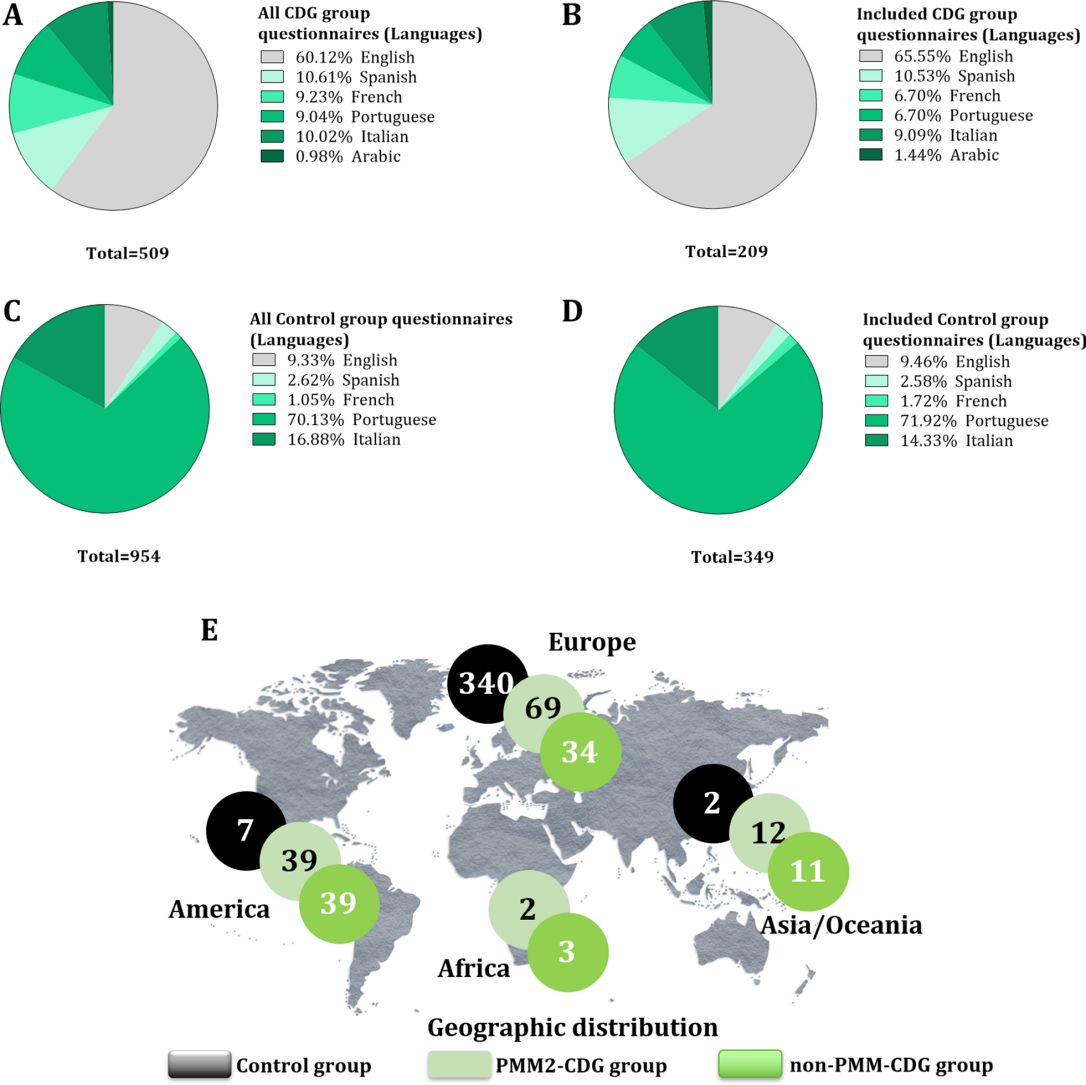
Language distribution (%) of all initiated and included ImmunoCDGQ (CDG group) and ImmunoHealthyQ (control group) questionnaires;** A) **All initiated questionnaires per language by the CDG group;** B)**; All included questionnaires per language in the CDG group;** C)** All initiated questionnaires per language by the control group;** D)** All included questionnaires per language in the control group;** E)** Geographical distribution of the CDG and control group participants

The ImmunoCDGQ (CDG group) and the ImmunoHealthyQ (control group) were initiated by 509 and 954 participants, respectively (Table 1, Fig. 4A, C). Participants had to read and agree to the electronic informed consent to proceed to the questionnaire. In the CDG group, 4 participants did not consent to participate whereas all control participants agreed to take part in the study. The average completion rate, i.e., the percentage of participants who completed the entire questionnaire was below 50% and equivalent between both groups, being of 43.4% (221 out of 509) in the CDG group and of 43.6% (416 out of 954) among controls (Table 1). What differed was the inclusion rate, i.e., the percentage of included participants following the filtering of the inclusion/exclusion criteria. Questionnaire inclusion rate was much higher in the CDG group (94.6%, 209 out of 221) compared to controls (83.9%, 349 out of 416) (Table 1). Reasons for excluding complete questionnaires among the CDG group included (i) unconfirmed or incomplete CDG diagnosis (n = 10) and (ii) strong suspicions of duplicated patient reporting (n = 2). In the control group 67 participants were excluded for having a genetic or chronic condition.

**Table 1:** Questionnaire completeness and inclusion rates among the and CDG (ImmunoCDGQ) and control (ImmunoHealthyQ) groups

	CDG group (ImmunoCDGQ)	Control group (ImmunoHealthyQ)
Nº of initiated questionnaires	509	954
Nº of completed questionnaires	221	416
Nº of included questionnaires	209	349
Completion rate (%, average)	43.4%	43.6%
Inclusion rate (%, average)	94.6%	83.9%

Regarding the questionnaire language distribution and representativeness, most of the initiated and included ImmunoCDGQ responses were in English, followed by Spanish and then Italian (Fig. 4A, B). Contrastingly, the most represented language in the ImmunoHealthyQ was Portuguese, followed by Italian and English (Fig. 4C, D). Regarding completion and inclusion rates of the different language versions, they varied not only between the control and CDG groups but also amongst themselves (Additional file 1: Tables S5 and S6).

The included 209 CDG and 349 control participants were from 31 and 12 countries, respectively. Complete participant demographics are detailed in [10] (Fig. 4E).

### ImmunoCDGQ has higher and more variable completion time commitments

The total number of items present in the ImmunoCDGQ and ImmunoHealthyQ was 58 and 56, respectively. However, of those only 52 and 50 were mandatory. The use of logic, as illustrated in Fig. 2, reduced the number of questions answered by participants, particularly in the absence of immune-related manifestations (Additional file 1: Figure S1). In addition and consequently to impacting the number of questions participants had to answer, conditional logic structing affected the time commitment required to complete the questionnaire. Among the included questionnaires in the CDG and control groups there were several differences. PMM2-CDG patients accounted for 58.4% (122 out of 209) of the included questionnaires. Hence and similarly to what was performed in the clinical data analysis [10], whenever found appropriate, the CDG group is further detailed and divided into two subgroups, henceforth referred to as: the PMM2-CDG and non-PMM2-CDG groups. The latter encompasses all included questionnaires describing patients with a CDG other than PMM2-CDG.

Overall and independently of the immune status of the participants (with or without immunological involvement, or even with an unsolved immune status), on average, the CDG group required more time to fill out the questionnaire (Additional file 1: Table S7). Moreover, CDG participants showed higher variation in their time commitments to complete the questionnaire. Among controls, not only was there higher homogeneity in terms of time investment, but there was also a clearer relationship between the number of questions participants answered and the time they took to complete the questionnaire. Control participants reporting infections required the most time while those without immunological involvement spent the least amount of time answering the questions (Additional file 1: Table S7).

In terms of the questionnaire language versions, the Portuguese and Spanish versions of the ImmunoCDGQ took longer to complete (on average), whereas in the ImmunoHealthyQ, it was the English version (Additional file 1: Table S8).

### A fifth of the ImmunoCDGQ participants opted to not complete the full questionnaire in one session

At the beginning of the questionnaire participants were alerted of the need/advantage of having blood and any other relevant immune-related lab results. Genetic testing results were also requested. Recognizing the amount of required underlying information, predicted time commitment and the burdensome lives of CDG patients and family caregivers, the option to allow participants to fill out the entire questionnaire in more than one go was enabled. In fact, the only mandatory deadline for questionnaire completion and submission was established by the study’s closing date. Hence, during that period participants could return to the questionnaire as many times as they felt necessary, completing it at their own pace. Twenty % of all the included CDG participants (42 out of 209) did not reply to the entire questionnaire in one session, taking—on average—21.8 days to fully complete it. Among these 42 CDG participants, 71.4% reported PMM2-CDG patients (30 out of 42). Noteworthy, in the CDG group, most of the participants who failed to complete the whole questionnaire in one session reported immunological involvement (57.1%, 24 out of 42). This trend was also present in both the PMM2-CDG and non-PMM2-CDG subgroups. Oppositely in the control group every participant filled out the entire questionnaire in one go (Table 2).

**Table 2:** Number of participants who did not complete the entire questionnaire in one session

Participant groups	Nº of participants not completing the questionnaire in one session	Nº of days to complete the questionnaire		Immune status		
		µ	SD	0	1	2
CDG group (Total)	42 out of 209 (20.0%)	21.8	23.5	13 (31%)	24 (57.1%)	5 (11.9%)
PMM2-CDG group	30 out of 122 (24.6%)	21.2	22.5	9 (30%)	17 (56.7%)	4 (13.3%)
Non-PMM2-CDG group	12 out of 87 (13.8%)	23.2	26.9	4 (33.3%)	7 (58.3%)	1 (8.3%)
Control group	0 out of 349 (0%)	NA				

Results are presented per participant group and according to the immunological status. Percentages (%) are shown

0, without immunological involvement; 1, with Immunological involvement; 2, unsolved immune status; NA, not applicable; SD, standard deviation

### Conditional logic structuring resulted in over a fourth of CDG and control participants having an immune status reassignment

Conditional logic structuring guided data provision, particularly the identification of immune-related manifestations affecting the CDG patient or healthy participant, as it allowed confirmation or refutation of the provided information. Participants were first confronted with a general question about immunological involvement, which stated “Has the CDG patient (or participant) ever had any immune system problem?” (Fig. 2). In 26.6% of CDG participants (56 out of 209) and 29.5% of the control group (103 out of 349) their immune status reporting in this first question was changed in follow-up and more specific questions. Hence, according to the defined criteria establishing the immune status (0 = without immunological involvement, 1 = with immunological involvement or 2 = unsolved immune status), these participants were reassigned to a category better suiting their reports. Both in the CDG (75%, 42 out of 56) and control (66%, 68 out of 103) groups, most of these participants firstly identified no immune-related problems but in follow-up questions, i.e., when confronted with specific immune-related manifestations, such as infections, described the presence of one or more of these clinical signs (58.9%—33 out of 56—of the CDG group and 68.9% among the controls—71 out of 103) (Table 3). In CDG, among the participants who shifted their immune status, although the highest number identified only allergies (48.5%, 16 out of 33) in subsequent questions, a considerable number reported infections too (36.4%, 12 out of 33). In the control group, 77.5% participants who had an immune status reassignment only described allergies (55 out of 71). Interestingly, in the PMM2-CDG group, besides the higher swing towards more participants being reclassified as having immunological involvement (from 20%—8 out of 40—to 50%, 20 out of 40), there was also a sizeable increase in those with an unsolved immune status (from 12.5%—4 out of 40—to 30%, 12 out of 40), i.e., participants without a defined immune status due to the lack of clear reporting on immune-related issues (Table 3). Concerning the questionnaire’s language versions, in the ImmunoCDGQ, the Portuguese and Italian versions were the ones with higher immune status alterations (54.5%, 6 out of 11, in both languages). In the ImmunoHealthyQ, the language versions with more immune status reclassifications were the English (36.4 9%, 12 out of 33) and Spanish (33.3%, 3 out of 9) (Additional file 1: Table S9).

**Table 3:** Number of study participants who had an immune status reassignment

Participant groups	Nº of participants whose immune status was reassigned	Immune status reported by participant (Initial)			Immune status following defined criteria application (Final)		
		0	1	2	0	1	2
CDG group (Total)	56 out of 209 (26.6%)	42 (75%)	9 (16.1%)	5 (8.9%)	9 (16.1%)	33 (58.9%)	14 (25%)
PMM2-CDG group	40 out of 122 (32.8%)	27 (67.5%)	8 (20%)	5 (12.5%)	8 (20%)	20 (50%)	12 (30%)
Non-PMM2-CDG group	16 out of 87 (18.4%)	15 (93.7%)	1 (6.3%)	0	1 (6.3%)	13 (81.2%)	2 (12.5%)
Control group	103 out of 349 (29.5%)	68 (66%)	23 (22.3%)	12 (11.7%)	25 (24.3%)	71 (68.9%)	7 (6.8%)

Number and percentages of participants whose answer to the first question about immunological involvement (highlighted in light red in Fig. 2) was different to the follow-up information provided in specific immune-related manifestations sections

0, without immunological involvement; 1, with Immunological involvement; 2, unsolved immune status

### Varied and multi-channel recruitment campaigns created stable and continuous participant engagement

Acknowledging the worldwide dispersion of the CDG community, strategic partnering with relevant CDG institutions and experts, and mapping of the CDG families preferred web-based platforms, particularly in social media, were performed. These measures promoted the development of tailored multi-channel and multi-stakeholder engagement and recruitment campaigns.

Three major phases can be distinguished for the social media strategies and campaigns: (i) Pre-ImmunoCDGQ launch; (ii) ImmunoCDG recruitment (divided into 3 sub-phases which include questionnaire launch and closing); (iii) ImmunoCDGQ result communication. In total, 73 different posts were published on various social media channels, namely Facebook, Twitter (448 followers), LinkedIN (1580 followers), Youtube (92 subscribers) and RareConnect (CDG Community, 339 members) between 18th September 2018 and 11th August 2020. On Facebook, both a public page (Sindrome CDG, 1951 Followers) and a closed group (CDG Global Alliance, 1163 members), the latter entirely made up of CDG families and professionals, were used to share information, project updates and results (Additional file 1: Tables S2, S3 and Additional file 2).

Table 4 summarizes the impact metrics of 4 social media campaigns (“10 Days = 10 CDG Immunology Facts”, “Why should CDG Families participate in the ImmunoCDGQ?”, “#ProtectCDG” and the result communication social media campaign) published on Facebook, Twitter, and LinkedIn. These campaigns are representative of and divided by the three-umbrella engagement phases (Table 4). Illustrative examples of posts disseminated in all these campaigns are shown in the Additional file 1: Figure S2.

**Table 4:** ImmunoCDGQ social media campaigns metrics

Social media (metrics)	Pre-launch	Recruitment		Result communication (5 posts)
	10 Days = 10 CDG Immunology Facts (11 posts)	*Why should CDG families participate in the ImmunoCDGQ?* (21 posts)	#ProtectCDG (6 posts)	
Facebook				
Nº of likes	112	208	61	53
Nº of shares	58	55	12	31
Nº of comments	0	6	1	4
Nº of people reached	3584	88,760	2919	2870
Nº of interactions	472	1064	349	363
Twitter				
Nº of likes	54	79	26	9
Nº of retweets	37	52	18	4
Nº of comments	0	0	1	1
LinkedIn				
Nº of likes	19	45	21	6
Nº of shares	0	2	0	0
Nº of comments	0	0	0	0

Listed social media campaigns are representative of the (i) pre-launch, (ii) recruitment and (iii) result communication published on Facebook (Sindrome CDG page), Twitter, and LinkedIn

Besides social media, e-mailing was continuously adopted throughout the engagement and recruitment phases. An important highlight was the short motivational emailing campaign which entailed a series of engagement messages distributed in 5 languages (Additional file 2). Conferences and meetings were also a pursued avenue, particularly during the pre-launch and result dissemination phases (Fig. 3).

**Fig. 5:**
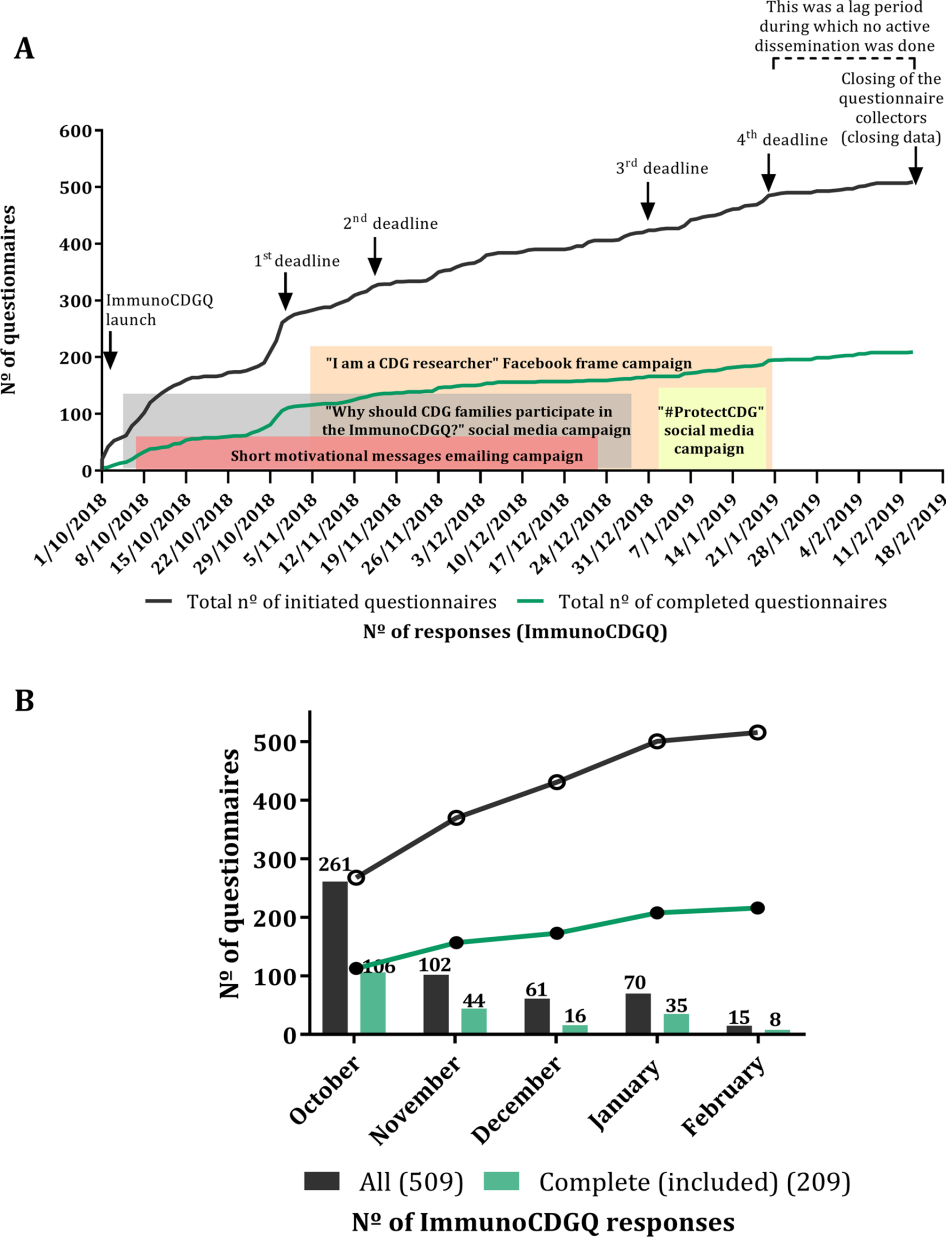
Evolution of the ImmunoCDGQ responses over time and their correlation with engagement campaigns; Legend: **A** Nº of ImmunoCDGQ responses (both initiated—in black—and completed—in green)—from the questionnaire launch to its closing. Response relationship with the main web-based engagement and recruitment campaigns and with announced study deadlines is depicted; **B** Monthly distribution of the nº of ImmunoCDGQ responses (both initiated—in black—and completed—in green) from the questionnaire launch till its closing

The crossing of answers’ evolution (of both initiated and included questionnaires) with the main web-based campaigns’ timelines reveals a consistent pattern of steady and continuous increase in the number of participants with steeper rises close to the announced ImmunoCDGQ deadlines. A total of 4 deadline extensions were created to stimulate participation (Fig. 5A). However, approximately 50.7% (106 out of 209) of all the included questionnaires were completed within the first month after the ImmunoCDGQ launch (Fig. 5B). Equivalent trends were registered in the different ImmunoCDGQ language versions (Additional file 1: Figure S4).

These results strongly suggest that the ImmunoCDGQ dissemination efforts retained high and continuous engagement levels of the CDG community, even though the recruitment rate had a clear relationship with the early stages of the project launch and the announcements of deadline extensions.

### Rare disease e-questionnaires are mostly internationally administered author-built tools supported by patient organizations in their web-based recruitment strategies

Through literature search and revision, 44 original studies were identified as meeting the defined inclusion/exclusion criteria (Additional file S1: Table S4 and Additional file 3). Yearly publication distribution revealed the oldest study being from 2001 and that 70.5% of these articles have been published since 2014 (31 out of 44). This evidenced the recentness of this field and methodologies.

Most studies were single disease focused (65.9%, 29 out of 44) and generally fell under a mixed category, as they combined clinical, HRQoL and/or information needs data collection (47.7%, 21 out of 44). Additionally, 36.4% solely collected medical information (16 out of 44), 11.4% HRQoL data (5 out of 44) and two studies focused exclusively on health-related information needs. Over half of the e-questionnaires (56.8%, 25 out of 44) were author-built, 36.4% (16 out of 44) mixed-built (combining author-built sections with existing tools) and 3 studies only employed existing questionnaires. As for the digital platforms used in the e-questionnaires’ development, they were often unnamed (47.7%, 21 out of 44). Still, a few platforms were identified, such as SurveyMonkey (5 out of 44), RedCap (3 out of 44) and Qualtrics software (2 out of 44). Oppositely, 84.1% of the studies reported ethical submission (37 out of 44).

Although 52.3% of the studies had an international scope (23 out of 44)—i.e., included participants from ≥ 2 countries, for most e-questionnaires either language was not disclosed (38.6% 17 out of 44) or they were only available in English (31.8%, 14 out of 44).

In 38.6% of the included projects targeted audiences were both patients and family members or caregivers (17 out of 44). Noteworthy, in 3 studies a control group was also recruited. Concerning the involvement of patients throughout the different stages of the project design, development, and dissemination, percentages greatly differed according to the phase. The stage where patient involvement was less prevalent and/or unreported was in the conceptualization phase (14 out of 44 studies). Contrastingly, the recruitment/dissemination stage was the one with the most frequent patient participation (95.5%, 42 out of 44). In line with this is the fact that, even though in most projects the recruitment was led by the research team their efforts were always supported by individual patients, patient leaders, POs and/or online patient communities (52.3%, 23 out of 44). Additionally, direct patient engagement in the development and the revision/piloting of the e-questionnaire occurred in 47.7% (21 out of 44) and 43.2% (19 out of 44), respectively.

E-questionnaires’ engagement and recruitment campaigns mainly explored the combinatorial use of diverse internet-based outlets and materials. Over 68% (30 out of 44) used social media networks, 61.4% utilized websites (27 out of 44) and 45.5% e-mail messages (20 out of 44). Among referred social media channels, Facebook was the most frequently mentioned (76.7%, 23 out of 30). Regarding the duration of the recruitment period, 4.5% of the questionnaires were open for participation for under one month (2 out 44), 43.3% between 1 to 6 months (19 out of 44), 22.7% recruited for 7 to 12 months (10 out of 44), and 18.2% admitted participants for more than 12 months (8 out of 44). The total numbers of respondents, completion and inclusion rates not only varied greatly but were also not uniformly described. Finally, what stood out was that only 3 studies described measures to divulge results among participants.

## Discussion

The ImmunoCDGQ development and recruitment strategies created a sustained engagement process that stretched from project ideation to result communication and involved multiple perspectives throughout the entire project. The recruitment of a control population is another innovative aspect of the present study, when compared to the literature [10]. Nevertheless, this study shares some commonalities with published studies applying similar methodologies, including the ImmunoCDGQ international outreach. This likely stems from the global distribution of RD patients, their generalized interest in contributing to research and the boundaryless qualities of internet-based research [13, 36]. Unlike a high number of existing studies which only used English questionnaire tools, the ImmunoCDGQ and its adaptation to the healthy population were translated into various languages.

E-questionnaire building with SurveyMonkey—a digital software also utilized in other analogous works [37–40]—permitted structuring the ImmunoCDGQ with conditional logic. This gave participants the chance to review their answers, while gradually confronting them with more specific immunology-related topics. These progressive steps assisted participants in providing more accurate information while also contributing to data analysis refinement. Moreover, offering participants’ the chance to progressively complete the questionnaire proved to be a valuable measure which was utilized by 20% of CDG participants. Various reasons could have influenced this CDG-specific behavior. Among them are the clinical complexity of CDG often associated with multi-disciplinary care, varied management therapies, medical emergencies, and special educational needs. These factors not only make clinical data reporting more demanding but add to the family juggling care and management burden [41].

Regardless of these adjustments and independently of the presence or absence of any immune-related manifestation, on average CDG participants consistently required more time to complete the questionnaire than the control group. More importantly, they showed a higher variation in terms of the questionnaire completion time. This could be related to the greater difficulties expressed by CDG family members and patients in answering the questionnaire, and due to the higher need to consult the provided immunology glossaries. Altogether these results simultaneously reinforce and frame the greater reporting of immunology-related information needs when compared to the control group [10].

Disease-related information seeking, and education are widespread RD community needs [42–45]. The differentiated and unmet information needs of CDG families, their interest in digital innovations, their use of social media and of POs to overcome information-related gaps have been documented and inspired the adopted ImmunoCDGQ engagement strategies [16, 41]. ImmunoCDGQ social media dissemination entailed the mapping of the most popular internet-based platforms and credible worldwide POs and professionals who assisted in the development of engagement materials. These bespoke procedures ensured more targeted and effective communication, abiding by the suggested social media-based research recruitment and educational material development best practices [32, 46]. Our results also clearly identified Facebook as the most successful social media channel amongst the CDG community members. This is probably related to the long withstanding existence of the closed “CDG Global Alliance” group created in August 2009. This group is perceived as a friendly, collaborative, and safe environment where families post questions and openly share their experiences. Moreover, this result is in accordance with the literature, showing Facebook as the most used social media channel in equivalent studies [47–51]. Still, the rise of other social media outlets, such as Instagram and Twitter, and the expressed interest of utilizing e-platforms as health education channels, underline the need and space to further explore these networks in web-based research projects [43].

Of note is that although the ImmunoCDGQ was open for participation for over 3 months, the highest number of responses occurred within the first month following the project launch. This could result from an anticipatory movement created by the pre-launch engagement and educational campaign, which instigated early family participation. A relevant portion of published e-questionnaire papers had a recruitment period lasting 1 to 6 months with only two cases—one of them a pilot study—recruiting for under a month [47, 52]. More data on response evolution in comparable e-questionnaires is warranted to better understand if this response rate is a common trend. These data would be relevant to guide recommendations on the duration of the recruitment period. Even though, a case-by-case evaluation would still be indispensable.

All in all, several figures illustrate the success of the implemented ImmunoCDGQ people-centric framework:

- Most participants reported a good understanding of the questionnaire content, rated the created immunology glossaries as useful as well as described participation as a comfortable and independent experience [10];
- The high inclusion rate of the completed CDG questionnaires;
- The large recruited patient cohort, which is, to date, the most international (31 different countries) and diverse (reporting 35 distinct CDG) published in CDG [10]. These results supplanted by far previous studies from our group that targeted the same audience [22]. Indeed, PMM2-CDG has an estimated incidence of 1:20 000, based on carrier frequency, with approximately 1200 patients known worldwide. Hence, our project was able to capture 10% of the PMM2-CDG global patient population.

### Study limitations

E-research, self-report approaches are not without pitfalls, namely lack of definition of ethical implications, lack of opportunity to clarify doubts among participants, and the possibility of malicious responses [53, 54]. To tackle these shortcomings, several safeguard and risk containing measures have been taken in this work to minimize such events, namely to:

- Have an e-consent form;
- Submit the project to an independent ethics committee—a procedure that is amply adopted by the research projects identified by our literature screening;
- Make available the contacts of the research team;
- Implement strict inclusion and exclusion criteria;
- Design informative, lay language resources that give participation instructions and explained difficult and technical concepts.

Online approaches can also exclude people with low e-health and/or digital literacy, and lack of access to the internet. Moreover, there is a possibility that the population sample included in this study represents a fringe of the CDG population who has higher education and literacy levels [37, 50, 55]. On the one hand, this can act as a limitation in the capturing of the broader immunology needs and management strategies. On the other hand, being the primary aim of the ImmunoCDGQ to collect clinical information, this potential participant bias could have ensured and increased the quality and validity of the collected data. Another aspect that would merit improvement is the possibility to establish an association between the number of recruited participants and each of the used recruitment media (social media, e-mailing or other).

### Future directions and recommendations

Considering the methodologies and results obtained in this and other published studies, some best practice proposals and future directions suggestions emerge:

1. Create a multistakeholder and multidisciplinary team. It should include patients/family members and professionals from diverse backgrounds who actively participate and guide decision-making in every stage of the project, starting from project ideation, conceptualization and extending to study development, piloting, and communication. Partnerships with POs may be particularly relevant to help identify patient (or family caregiver) leaders whose views and opinions are representative of the wider community and not exclusively focused on their own personal experience. Additionally, strategic patient partnerships can be particularly useful for recruitment purposes;
2. Map the needs and preferred communication channels of the targeted audience. The ImmunoCDGQ pre-launch campaign served empowerment and engagement purposes—addressing recognized gaps—but also aimed at anticipating the CDG community and building a momentum around the study. Hence, early engagement, definition and incorporation of the study audience needs, behaviors and preferences will aid in optimizing and tailoring strategies;
3. Diversify the educational and engagement resources. Experiment with different formats, such as videos, animated presentations and/or detailed written documents. It is important to acknowledge the distinct preferences but also the varying levels of literacy (digital, health) of the potential participants;
4. Identify and estimate the global outreach of the study and translate as much as possible, including questionnaire tools and other materials. This will not only influence the number of participants but the trustworthiness and quality of the gathered data, as well as improve participant experience;
5. Collect metrics. Take advantage of the various existing quantitative digital tools, some directly embedded in web-based platforms (e.g., Facebook statistics), to analyze and report the outreach and impact of the study;
6. Include result communication in the study plan from the start. The CHERRIES checklist—which is a central guideline in e-survey development and reporting—is amiss in this respect. Also, researchers tend to resort to more common communication channels, mainly scientific papers and conferences. However, these communication vehicles can exclude study participants, hence not following the recommendations of effective, accessible, and inclusive science communication. Consequently, it is important to give the audience periodic study updates, including on data analysis. In this respect too, the creation and sharing of lay language, comprehensive and appealing materials are fundamental to reach wider audiences.

Currently, we are working on developing a live section dedicated to CDG and immunology at the World CDG Organization platform. The WorldCDG.org is a central information digital hub directed at all members of the CDG community. The CDG and immunology section will harbor updates related to the ImmunoCDGQ results, such as information on upcoming associated studies and results. Moreover, the informative and educational resources created on this topic and important data generated by other authors will be included. The fact that this is a live repository allowing for constant updates, recognizes and helps convey the ever-evolving nature of scientific discoveries.

## Conclusions

Web-based platforms have potentiated people-centric research, especially encompassing clinical and HRQoL data collection in RDs. Besides providing tools for gathering and storing data, the internet concomitantly supports digital solutions for the creation and distribution of engagement, educational and recruitment materials [56]. The ImmunoCDGQ implemented a full people-centric methodology, involving multiple CDG community members, from project ideation to its development, and dissemination. The diverse and inclusive strategies deployed resulted in permanent CDG patient and family engagement and high participation. The impressive participation results are also in line with the described collaborative and even proactive attitude towards research found among CDG families [16]. Additionally, the educational angle explored in this study resulted in an important empowerment outcome.

Although integrated e-people centric research is still recent and with substantial room for growth and improvement, the progress that has been made in this field is undeniable. Indisputable is also the pivotal part POs have been playing and the wildcard accelerator that the COVID-19 pandemic has been to online research and care methodologies [57, 58]. These insights and advances can be leveraged to promote more and better research projects that address not only clinically relevant topics, but also the real needs and concerns of those living daily with these diseases.

## Supplementary Information

**Additional file 1:** Figures (Figure S1–S4) and Tables (Table S1–S9) to the main manuscript results.

**Additional file 2:** Metrics and target audience of ImmunoCDGQ dissemination and result communication social media and emailing campaigns.

**Additional file 3:** Characterization of the papers included in the literature revision. Specific data parameters were extracted from the papers included in the comparative literature revision included in this study.

## Data Availability

All data generated or analysed during this study are included in this published article [and its Additional files 1–3].

## Abbreviations

CDG: Congenital Disorders of Glycosylation
CDG & Allies-PPAIN: CDG & Allies—professionals and patient association international network
CHERRIES: Checklist for reporting results of internet E-Surveys
ERNs: European Reference Networks
HRQoL: Health-related quality of life
ImmunoCDGQ: Immunology and CDG questionnaire
ImmunoHealthyQ: Immunology questionnaire for the general “healthy” population
POs: Patient organizations
RD: Rare disease
