# Supplementary material for: The road to successful people-centric research in rare diseases: the web-based case study of the Immunology and Congenital Disorders of Glycosylation questionnaire (ImmunoCDGQ)
Source: Orphanet J Rare Dis. 2022 Mar 24;17:134. doi: 10.1186/s13023-022-02286-w (PMC8944152; doi:10.1186/s13023-022-02286-w)
Supplement: Supplementary file 1 — Additional file 1: Figures (Figure S1–S4) and Tables (Table S1–S9) to the main manuscript results. [file 13023_2022_2286_MOESM1_ESM.doc]

Supplementary table 1. Checklist for reporting results of internet E-Surveys (CHERRIES) for the Immunology and CDG questionnaire (ImmunoCDGQ) and its adapted version to the general “healthy” population (ImmunoHealthyQ).

| **Item category** | **Checklist item** | **This study** |
| --- | --- | --- |
| Design | Describe survey design | Target population(s): CDG and healthy individuals of all ages. Targeted respondents: CDG patients and caregivers (>18 years-old) and healthy people (general population,>18 years-old) (control group) |
| IRB approval and informed  consent process | IRB approval | This study was approved by Hospital Egas Moniz ethical committee [registration number 20170700050,  April 23rd, 2018]. |
|  | Informed consent | Electronic informed consent was obtained for every participant. Additionally, detailed and essential information on the project was also made available online on webpages specifically created for the project harboring diverse informative materials and resources: (<https://www.researchcdg.com/immunocdgq.html> and <https://www.researchcdg.com/immunoq.html>) |
|  | Data protection | Participant data was anonymous, and a coding system was created by the research team for data analysis purposes. Raw data was only made available to the main study researchers. All data collected and processed has been securely and stored on servers of NOVA Science and Technology School, NOVA University Lisbon (FCT-UNL) and its use and access are in accordance with Regulation (EU) 2016/679 of the European Parliament and of the Council of 27 April 2016 (RGPD), national protection laws and other relevant legislation and recommendations. |
| Development and pre-testing | Development and testing | Questionnaire developed using the SurveyMonkey platform. Questionnaire content and structure were developed based on literature data and input from two advisory boards (a CDG family and a medical/scientific expert boards). Two pilot phases involving CDG caregivers and medical experts were undertaken. For the control group, an adapted version of the questionnaire was administered. |
| Recruitment process and  description of the sample  having access to the  questionnaire | Open survey versus closed  survey | Open questionnaire using targeted and bespoke recruitment strategies/channels |
|  | Contact mode | E-mail, Skype, and telephone contact from the leading researcher was made available. Also, contact with participants through a closed Facebook group was facilitated. |
|  | Advertising the survey | The questionnaire was disseminated using several web-based platforms and software (Facebook, Twitter, RareConnect, e-mailing lists, Webpages, Skype meetings) and CDG scientific meetings. For the control group, e-mailing lists of universities and research institutions were the main utilized channels. |
| Survey administration | Web/E-mail | Links to the questionnaire (in the different languages versions) were posted on web-based platforms and shared by e-mail as well. |
|  | Context | All the web-platforms used in the questionnaire dissemination had as the main (and, in some cases exclusive) audience the CDG community, e.g., CDG Global Alliance (closed Facebook group) and RareConnect CDG Community group. Also, these were international channels which encompassed CDG Community members (mostly CDG parents and family caregivers) and which are frequently used by their members as health discussion forums. Importantly, a previously published study by our group had used these same channels and obtained good participation and engagement (**doi**: 10.1007/8904_2018_121). |
|  | Mandatory/voluntary | Voluntary questionnaire. |
|  | Incentives | No incentives were offered other than sharing of results with participants. |
|  | Time/date | The questionnaire was open for participation for:  - the CDG group from 01/10/2018 to 15/02/2019, and;  - the control group from 09/05/2019 to 24/06/2019. |
|  | Randomization of items or questionnaires | No randomization applied. |
|  | Adaptive questioning | The SurveyMonkey conditional logic feature was applied in several key questions to guide participation and reduce participant burden. Hence adaptive questioning was repeatedly used in this questionnaire. |
|  | Number of items | The total number of survey items was 58. However, due to the use of adaptive questioning, the number of items replied to by any participant was always fewer than that (maximum number of items replied by any participants being 55).  In the control group, the total number of items was 56, but the maximum number of items replied by any participant was 53. |
|  | Number of screens (pages) | As with the number of items, the number of screens was also variable, with the maximum number being 22 screens for the CDG group and 20 for the control group. |
|  | Completeness check | All, but the three final questions (dedicated to exploring the understandability of the questionnaire) were mandatory. Completeness checks were done with the help of SurveyMonkey filters after survey completion. Additionally, all questions add an “I don’t know” or “Not applicable” answer option to avoid forced choice. |
| Response rates | Review step | Respondents were able to review and change their answers while completing the e-survey by clicking on the preview button. |
|  | Unique site visitors | Unique site visitors were determined by IP addresses. |
|  | View rate (ratio of unique  survey visitors/unique site  visitors) | Due to the activation of the IP ID restriction, all the visitors were unique. |
|  | Participation rate (ratio of  unique visitors who agreed to  participate/unique first  survey page visitors) | 99 % (504 out of 509) for the CDG group and 100% (954 out of 954) for the control group |
|  | Completion rate (ratio of users  who finished the survey/users  who agreed to participate) | 43.8% in total (221 out of 504) for the CDG group and 43.6 % (416 out of 954) for the control group |
| Preventing multiple entries  from the same individual | Cookies used | SurveyMonkey’s cookie system |
|  | IP check | Repeated access from the same IP address was blocked by activation of the SurveyMonkey option “the questionnaire cannot be answered several times from the same device” |
|  | Log file analysis | No additional technical measures were undertaken to prevent duplicate entries. However, after raw data was automatically retrieved in excel format using SurveyMonkey data extraction features, all entries were screened by the lead researcher to rule out any duplicate reports of the same patient. When in doubt both reports were excluded. Additionally, the following inclusion/exclusion criteria were uniformly applied: Only CDG patients with confirmed and complete CDG diagnosis were included. For the control group, only participants not reporting chronic/genetic conditions were included. All participants failing to complete all relevant clinical sections were eliminated from further analysis. |
|  | Registration | NA |
|  | Handling of incomplete  questionnaires | Only questionnaires with all the mandatory clinical sections completed were analyzed. |
|  | Questionnaires submitted with  an atypical timestamp | A completion time cut-off was not established nor considered as an exclusion criterium. |
|  | Statistical correction | No weighting of items or propensity scores were adopted as they were inapplicable to our questionnaire. |

Supplementary table 2. Resources and materials created and disseminated during the i) ImmunoCDGQ Pre-launch; ii) Recruitment, and; iii) Result communication campaigns.

| **Type of resource** | **Title** | **Short description** | **Available languages** |
| --- | --- | --- | --- |
| **i)Pre-launch campaign** | | | |
| **PDF document - Extended version of the consent form** | Immunology CDG Questionnaire (ImmunoCDGQ) for CAREGIVERS & PATIENTS Informed Consent – EXTENDED VERSION | It is a document explaining the project: context, aims, team, benefits/harms, contacts | ENG, PT, FR, ES, IT |
| **PowerPoint presentation** | ImmunoCDGQ_slide deck | This is a slide deck with 15 slides explaining the project and which was shared with CDG families, patient associations, and professionals | ENG |
| **PowerPoint presentation** | ImmunoCDGQ_slide deck (short) | This is a slide deck with 9 slides explaining the project and which was shared with CDG families, patient associations, and professionals | ENG |
| **Prezi presentation** | Immunology Basics | A short Prezi presentation explaining basic immunology concepts | ENG |
| **Prezi presentation** | ImmunoCDGQ: Tips for a smooth participation | A short Prezi presentation explaining some technical aspects of the ImmunoCDGQ and giving tips | ENG |
| **Patient-friendly guide** | Immunological involvement in CDG | A lay language guide offering information and guidance on immune-related problems in CDG | ENG, PT, ES |
| **Scientific revision of the literature:** | Immunological aspects of congenital disorders of glycosylation (CDG): a review | Review of the literature done by our group | ENG |
| **Mini-glossaries** |  | There are 8 mini-glossaries, each dedicated to a specific topic related with CDG and immunology | ENG, PT, FR, ES, IT, AR |
| **ii) Recruitment campaigns** | | | |
| **Video** | Why should CDG Families participate in the ImmunoCDGQ français | La clinicienne et maman CDG Oriane Hostache explique pourquoi elle pense que les patients et les soignants CDG devraient prendre part à ce projet de recherche unique. | FR |
| **Video** | Why should CDG Families participate in the ImmunoCDGQ italiano | La mamma e avvocato Tatiana Rijoff spiega perché pensa che i pazienti e i caregivers CDG dovrebbero prendere parte a questo progetto di ricerca unico e molto importante -Questionario sull'Immunologia CDG per Pazientes & Caregivers "ImmunoCDGQ". | IT |
| **Video** | Why should CDG Families participate in the ImmunoCDGQ Dorinda | Dr Dorinda da Silva tells you why she thinks the ImmunoCDGQ is an important research project that you should join! | ENG |
| **Video** | Why should CDG Families participate in the ImmunoCDGQ ES | Sandra, la mama de Martim, explica como TODAS las familias CDG pueden contribuir para avanzar la investigación en el campo de la inmunología. Anímate y participa! | ES |
| **Video** | Why should CDG Families participate in the ImmunoCDGQ Morgan 1 | Morgan, CDG patient and advocate shares her views on the importance of the CDG Community participating in this unique project - the Immunology and CDG Questionnaire (ImmunoCDGQ) for Patients & Caregivers. | ENG |
| **iii) Result communication campaigns** | | | |
| **Magazine article** | Patients as partners - paving the way for scientific discoveries Rare Revolution Magazine article | Article based on an interview | ENG |
| **Video** | New Insights into Immunological Involvement in Congenital Disorders of Glycosylation (CDG) from a People-Centric Approach | Video summarizing the main conclusions of the scientific paper published describing the medical/scientific results of the project using simple and accessible language | ENG |
| **Posters** |  | Presented at conferences | ENG |
| **PPT presentations** |  | Presented at conferences | ENG |

Legend: AR – Arabic; ENG – English; ES – Spanish; FR – French; IT – Italian; PT – Portuguese

Supplementary table 3. List of conferences in which the study was promoted, explained and communicate during the i) ImmunoCDGQ Pre-launch, and ii) Result communication campaigns.

| **Conference/symposium** | **Venue** | **Conference date** | **Target audience(s)** | **Type of material presented** |
| --- | --- | --- | --- | --- |
| **i)Pre-launch campaign** | | | | |
| **USA CDG Family Conference** | San Diego, USA | 23-25 February 2018 | CDG Families | Oral presentation |
| **Spanish CDG Family Meeting** | Madrid, Spain | 21 April 2018 | CDG Families | Oral presentation |
| **Nordic CDG Family Meeting** | Stockholm, Sweden | 1-2 September 2018 | CDG Families | Oral presentation |
| **Italian CDG Family Meeting** | Italy | 23 November 2018 | CDG Families | Oral presentation |
| **Dutch CDG Family Meeting** | Netherlands | 31 January 2019 | CDG Families | Oral presentation |
| **ii)Result communication campaigns** | | | | |
| **Voice of the Customer Colloquium** | Aveiro, Portugal | 29-30 November 2018 | Scientists of various areas and fields | Oral presentation |
| **Ciência'19 meeting** | Lisbon, Portugal | 8-10 July 2019 | Scientists of various areas and fields | Poster presentation |
| **4th World CDG Conference for Families and Professionals** | Lisbon, Portugal | 26-27 July 2019 | CDG Families and Professionals | Oral presentation |
| **GluPor 13** | Porto, Portugal | 3-5 September 2019 | Scientists and clinicians of various areas and fields | Poster presentation |
| **1st National Rare Disease Congress (Portuguese)** | Sintra, Portugal | 31 October - 3 November 2019 | Clinicians, scientists and advocates from various areas and fields | Poster and oral presentation |
| **USA CDG Family Conference** | San Diego, USA | 28 February - 1 March 2020 | CDG Families | Oral presentation |
| **NOVA Health Conference** | Virtual event | 9 October 2020 | Scientists of various areas and fields | Poster presentation |
| **Society of Glycobiology meeting** | Virtual event | 9-12 November 2020 | Scientists and clinicians from the glycobiology field | Poster presentation |
| **SPDMI International Symposium** | Virtual event | 11-13 November 2020 | Scientists and clinicians from the metabolic field | Poster and oral presentation |

Supplementary table 4. Keyword combinations and applied study inclusion and exclusion criteria in literature screening. Databases (PubMed/Medline and Google Scholar) were searched using these keyword combinations from 16^th^ December 2019 to 12^th^ February 2021 to retrieve indexed and grey literature, respectively. In a first phase, a set of keywords were used, however, when the article reading and selection began, many of the retrieved papers were out of the scope. Hence, once the selection of these papers was concluded, a second search using additional keyword combinations was performed. The same inclusion/exclusion criteria were applied to filter the papers obtained using the first and second keyword combinations.

| **First keyword combination** | | | **Inclusion/ exclusion criteria** | **Second keyword combination** | | |
| --- | --- | --- | --- | --- | --- | --- |
| **Communication strategies** | AND | Rare diseases | **Exclusion criteria:**   - Reviews; - Articles not in English; - Articles not about Rare Diseases (diseases were considered rare according to Orphanet and GARD definition); - Full text not available - Surveys/questionnaires conducted and/or disseminated without the use of web-based methods (e.g., telephonic or postal distribution) - Surveys exclusively directed at healthcare professionals or focusing on healthcare professionals needs/perspectives - Caregiver surveys solely measuring caregiving-related aspects (e.g., Caregiver Burden), i.e. not directly related to the rare disease patient or to the impact of the disease on the patient; - Semi-structured interviews, focus groups and studies that only apply qualitative methods: - Studies directed at patient associations and not at single patients; - Studies employing the Delphi method; - Studies focusing only on determining research/treatment priorities - Unclear/unspecified content of the questionnaire (for e.g., only when the effectiveness of different recruitment strategies is presented with no reference to the topics analyzed by the questionnaire itself)   **Inclusion criteria:**   - Electronic surveys/questionnaires addressing, at least one of the following topics: clinical research, quality of life and/or health/disease information needs, and developing web-based dissemination and recruitment strategies; - Original research (including papers, MSc’s and PhD thesis, reports) | internet research | AND | Rare disease |
| **Research participation** |  | Patient communities |  | internet recruitment |  | Rare Patient Communities |
| **Communication campaigns** |  |  |  | Facebook |  |  |
| **Research engagement** |  |  |  | Twitter |  |  |
| **Empowerment campaigns** |  |  |  | RareConnect |  | Rare disease patient association |
| **Empowerment strategies** |  |  |  | Online patient community |  |  |
| **Empowerment needs** |  |  |  |  |  |  |
| **Information campaigns** |  | Patient associations |  | Email/E-mail recruitment |  |  |
| **Information needs** |  |  |  |  |  |  |
| **Patient involvement in research** |  |  |  |  | | |
| **Patient centric research** |  |  |  |  |  |  |
| **Patient centered research** |  |  |  |  |  |  |
| **Patient reported outcomes in immunology** |  |  |  |  |  |  |
| **Social media engagement** |  |  |  |  |  |  |
| **Social media research** |  |  |  |  |  |  |
| **Social media recruitment campaigns** |  |  |  |  |  |  |
| **Questionnaire-based research** |  |  |  |  |  |  |
| **Survey-based research** |  |  |  |  |  |  |
| **Immunology Questionnaire-based research** |  |  |  |  |  |  |
| **Immunology Survey-based research** |  |  |  |  |  |  |

Supplementary table 5. Questionnaire completeness and inclusion among the CDG group (ImmunoCDGQ).

|  | ENG | | ES | IT | PT | FR | AR |
| --- | --- | --- | --- | --- | --- | --- | --- |
| Nº of initiated questionnaires | 306 |  | 54 | 51 | 46 | 47 | 5 |
| Nº of completed questionnaires | 140 | 23 | | 23 | 17 | 15 | 3 |
| Nº of included questionnaires | 137 | 22 | | 19 | 14 | 14 | 3 |
| Completion rate (%, average) | 45 % | 41 % | | 37 % | 30 % | 30 % | 60 % |
| Inclusion rate (%, average) | 98 % | 96 % | | 83% | 82 % | 93 % | 100 % |

Legend: AR – Arabic; ENG – English; ES – Spanish; FR – French; IT – Italy; PT - Portuguese

|  | ENG | | ES | IT | PT | FR |
| --- | --- | --- | --- | --- | --- | --- |
| Nº of initiated questionnaires | 89 |  | 25 | 161 | 669 | 10 |
| Nº of completed questionnaires | 39 | 11 | | 59 | 300 | 7 |
| Nº of included questionnaires | 33 | 9 | | 50 | 251 | 6 |
| Completion rate (%, average) | 44 % | 44 % | | 37 % | 45 % | 70 % |
| Inclusion rate (%, average) | 85 % | 82 % | | 85 % | 84 % | 86 % |

Supplementary table 6. Questionnaire completeness and inclusion among the control group (ImmunoHealthyQ).

Legend: ENG – English; ES – Spanish; FR – French; IT – Italy; PT - Portuguese

**With Immunological involvement**

**Without immunological involvement or unsolved immune status**

**Min. number of questions answered**: 24

*(only 21 mandatory)*

**Nº of infection-related questions**:

22

**Min. number of questions answered**: 27

*(only 24 mandatory)*

**Max. number of questions answered:** 33

*(only 30 mandatory)*

**Max. number of questions answered:**

55

*(with only 52 being mandatory)*

**Nº of allergies/**

**autoimmunity-related questions**:

3

*For each section*

**CDG group**

**A**

**With Immunological involvement**

**Without immunological involvement or unsolved immune status**

**Min. number of questions answered**: 17

*(only 14 mandatory)*

**Nº of Infection-related questions**:

25

**Min. number of questions answered**: 22

*( only 19 mandatory)*

**Max. number of questions answered:** 24

*(only 21 mandatory)*

**Max. number of questions answered:**

53

*(only 50 being mandatory)*

**Nº of allergies/**

**autoimmunity-related questions**:

6

*For each section*

**Control group**

**B**

Supplementary figure 1. Scheme of the number of items answered by participants reporting and not reporting any immune-related manifestations, both in A) the ImmunoCDGQ (CDG group) and B) the ImmunoHealthyQ (control group). These number differences arise from the conditional logic applied to the development of the questionnaire. Additionally, the differences between the control and CDG group result from the necessary adaptions performed to make the questionnaire more adequate for the general “healthy” population in which CDG specific questions were omitted.


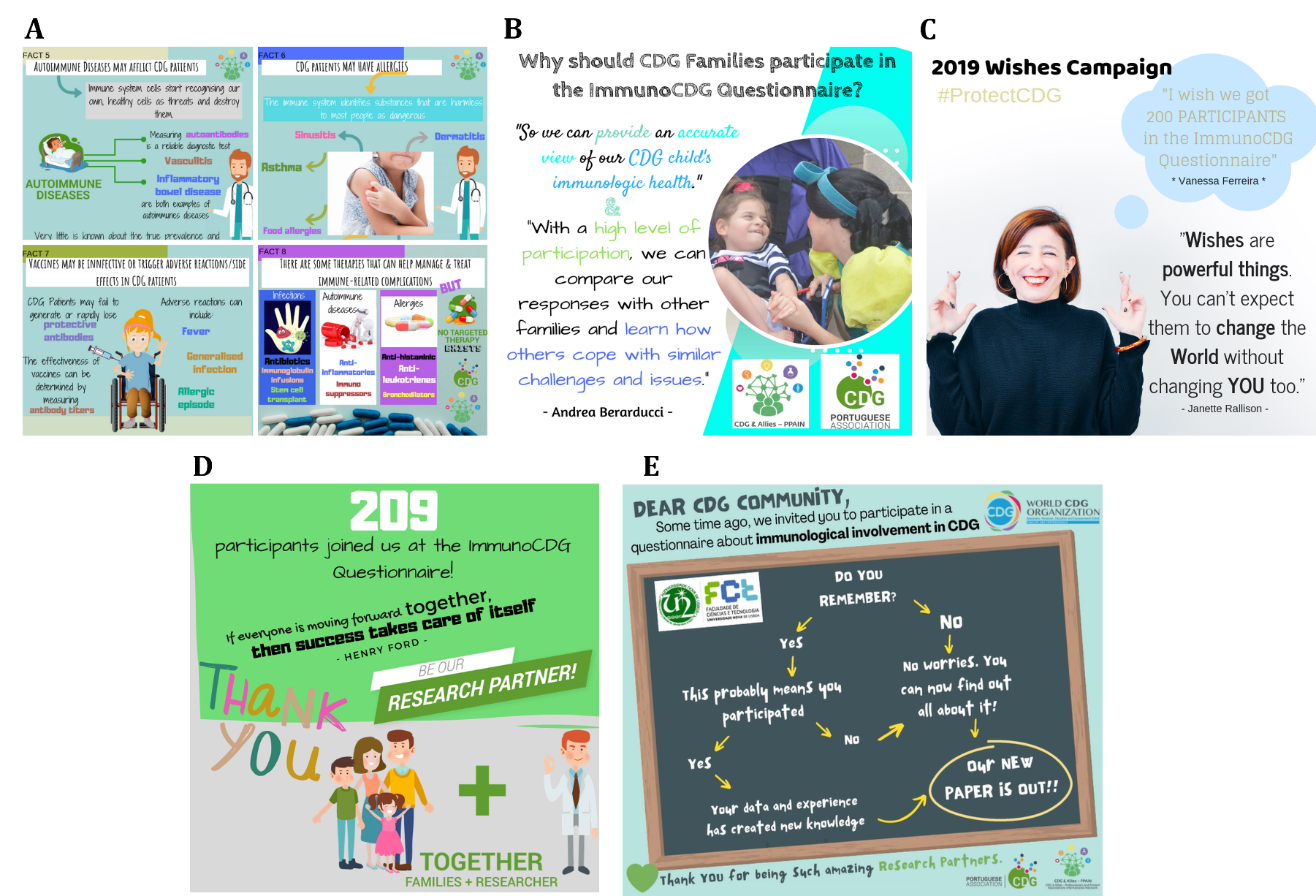


Supplementary figure 2. Examples of social media posts used in the ImmunoCDGQ engagement and recruitment campaigns. A) “10 Days = 10 CDG Immunology Facts”; B) “Why should CDG Families participate in the ImmunoCDGQ?”; C) “#ProtectCDG”; D) “Thank you” and other example of the E) Result communication campaigns.


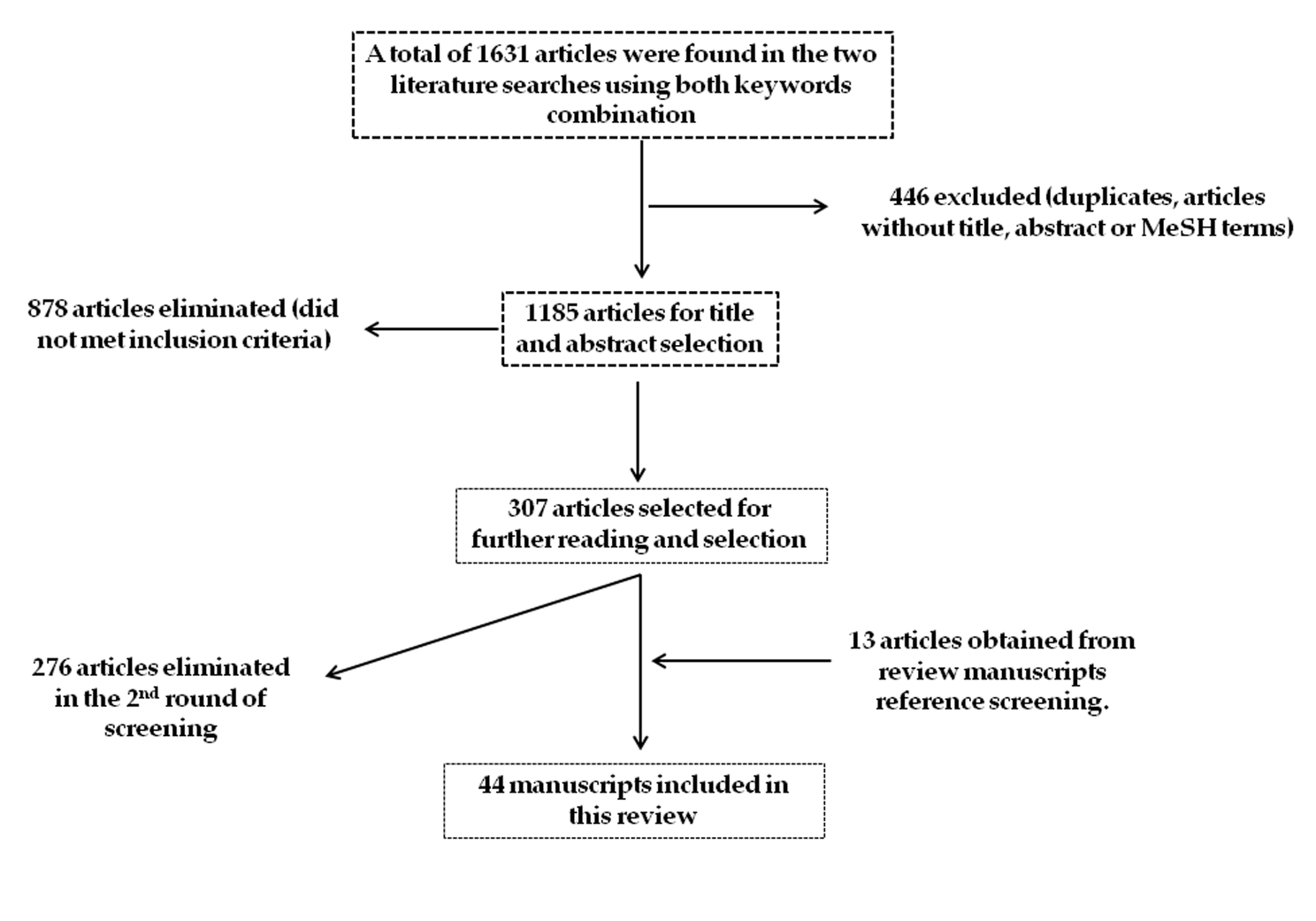


Supplementary figure 3. Flowchart of the paper selection process. Phases of paper identification and selection, from database search to the application of the inclusion/exclusion criteria.

Supplementary table 7. Questionnaire completion time per participant group and according to the immunological status. Participants not replying to the entire questionnaire at once were excluded from this analysis.

| Participant **group** | | | | Time of completion(all participants) | | |  | With immunological involvement | | | | | | Without immunological involvement | | | |
| --- | --- | --- | --- | --- | --- | --- | --- | --- | --- | --- | --- | --- | --- | --- | --- | --- | --- |
|  |  |  |  |  |  |  | Time of completion(With infection, min) | | | | Time of completion(Without infection, min) | | | | Time of completion (min) | | |
|  | | | | µ (min) | SD (min) | Nº participants | µ (min) | | SD (min) | Nº participants | µ (min) | SD (min) | Nº participants | | µ (min) | SD (min) | Nº participants |
| CDG group (Total,209) | | | | 33.6 | 29.3 | 167 | 36.5 | | 31.9 | 103 | 26.5 | 23.1 | 25 | | 29.6 | 26.2 | 37 |
|  |  | PMM2-CDG group (122/209) | | 33.6 | 32.7 | 92 | 36.6 | | 38.3 | 52 | 22.4 | 10.7 | 13 | | 32.2 | 29.6 | 23 |
|  |  | | Non-PMM2-CDG group (87/209) | 33.6 | 24.7 | 75 | 36.3 | | 24.0 | 51 | 30.9 | 31.6 | 12 | | 25.1 | 19.4 | 14 |
| Control group(Total, 349) | | | | 12.2 | 13.8 | 349 | 19.5 | | 13.5 | 53 | 11.8 | 13.4 | 129 | | 10.3 | 13.7 | 167 |

Legend: min- minutes; SD – Standard deviation

Supplementary table 8. Questionnaire completion time per language version and according to the immunological status. Participants not replying to the entire questionnaire at once were excluded from this analysis.

| **ImmunoCDGQ/**  **ImmunoHealthyQ: language versions** | | **Time of completion**  **(all participants)** | | | **With immunological involvement** | | | | | | **Without immunological involvement** | | |
| --- | --- | --- | --- | --- | --- | --- | --- | --- | --- | --- | --- | --- | --- |
|  |  |  |  |  | **Time of completion**  **(With infection)** | | | **Time of completion**  **(Without infection, min)** | | | **Time of completion (min)** | | |
| **CDG Group** |  | µ (min) | SD (min) | Nº participants | µ (min) | SD (min) | Nº participants | µ (min) | SD (min) | Nº participants | µ (min) | SD  (min) | Nº participants |
|  | English | 30.6 | 22.2 | 117 | 31.4 | 19 | 76 | 26.5 | 23.1 | 18 | 31.4 | 29.6 | 18 |
|  | Spanish | 41 | 58.4 | 16 | 66.8 | 85 | 6 | 28.2 | 13.4 | 5 | 15.3 | 2.5 | 3 |
|  | French | 29.7 | 11.2 | 9 | 32.4 | 7.8 | 7 | NA | NA | 0 | 20 | 19.8 | 2 |
|  | Portuguese | 50.5 | 37.9 | 11 | 60.3 | 44.6 | 6 | NA | NA | 1 | 38.5 | 32.3 | 4 |
|  | Italian | 39.8 | 33.0 | 11 | 49.3 | 42.6 | 6 | NA | NA | 1 | 27.0 | 11.8 | 3 |
|  | Arabic | 36 | 32.1 | 3 | 44.0 | 44.1 | 2 | NA | NA | 0 | NA | NA | 1 |
| **Control Group** |  | | | | | | | | | | | | |
|  | English | 14.8 | 21.8 | 33 | 15.0 | 4.2 | 4 | 15.3 | 15.4 | 9 | 14.5 | 26.4 | 20 |
|  | Spanish | 11.2 | 10.4 | 9 | NA | NA | 1 | 13.0 | 11.4 | 4 | 10.0 | 12.2 | 4 |
|  | French | 13.0 | 5.4 | 6 | NA | NA | 1 | 12.5 | 6.4 | 2 | 13.3 | 7.2 | 3 |
|  | Portuguese | 12.3 | 12.7 | 251 | 20.8 | 14.9 | 41 | 12.0 | 14.2 | 102 | 9.5 | 8.6 | 100 |
|  | Italian | 10.1 | 13.3 | 50 | 15.8 | 5.8 | 5 | 8.2 | 2.2 | 13 | 10.0 | 16.4 | 32 |

Legend: min – minutes; NA – Not applicable; SD – Standard deviation

Supplementary table 9. Number of study participants who had an immune status reassignment per language version. Number and percentages of participants whose answer to the first question about immunological involvement (highlighted in light red in Figure 2) was different to the follow-up information provided in specific immune-related manifestations sections.

| **ImmunoCDGQ/**  **ImmunoHealthyQ: language versions** | | **Nº of participants whose immune status was reassigned** | | **Immune status reported by participant (Initial)** | | | | | | **Immune status following defined criteria application (Final)** | | | | |
| --- | --- | --- | --- | --- | --- | --- | --- | --- | --- | --- | --- | --- | --- | --- |
|  |  |  |  | 0 | | 1 | | 2 | | 0 | 1 | | 2 | |
| CDG group | English | 36 out of 177 (20.3 %) | | 8  (22.2 %) | | 25  (69.4 %) | | 8  (22.2 %) | | 3  (8.3%) | 5  (13.9 %) | | 25  (69.4 %) | |
|  | Spanish | 5 out of 16 (31.3 %) | | 1 (20 %) | | 4 (80 %) | | 1 (20 %) | | 0 | 0 | | 2 (40 %) | |
|  | French |  | 2 out of 9 (22.2 %) | 0 | 2  (100 %) | | 0 | | 1 (50 %) | | | 1 (50 %) | | 0 |
|  | Portuguese |  | 6 out of 11 (54.5 %) | 3  (50 %) | | 3  (50 %) | | 0 | | 2  (33.3 %) | 2  (33.3 %) | | 2  (33.3 %) | |
|  | Italian |  | 6 out of 11 (54.5 %) | 4  (66.7 %) | | 1  (16.7 %) | | 1  (16.7 %) | | 2  (33.3 %) | 3  (50 %) | | 1  (16.7 %) | |
|  | Arabic |  | 1 out of 3 (33.3 %) | 0 | | 1 (100 %) | | 0 | | 0 | 0 | | 1 (100 %) | |
|  |  | | | | | | | | | | | | | |
| Control group | English |  | 12 out of 33 (36.4 %) | 1  (8.3 %) | | 7  (58.3 %) | | 4  (33.3 %) | | 8  (66.7 %) | 4  (33.3 %) | | 0 | |
|  | Spanish |  | 3 out of 9 (33.3 %) | 3 (100 %) | | 0 | | 0 | | 0 | 3 (100 %) | | 0 | |
|  | French |  | 0 out of 6 (32.8 %) | NA | | | | | | | | | | |
|  | Portuguese |  | 75 out of 251 (29.9 %) | 56  (74.7 %) | | 12  (16 %) | | 7  (9.3 %) | | 13  (17.3 %) | 55  (73.3 %) | | 7  (9.3 %) | |
|  | Italian |  | 4 out of 50 (8 %) | 0 | | 4 (100 %) | | 0 | | 4 (100 %) | 0 | | 0 | |

Legend: NA – Not applicable


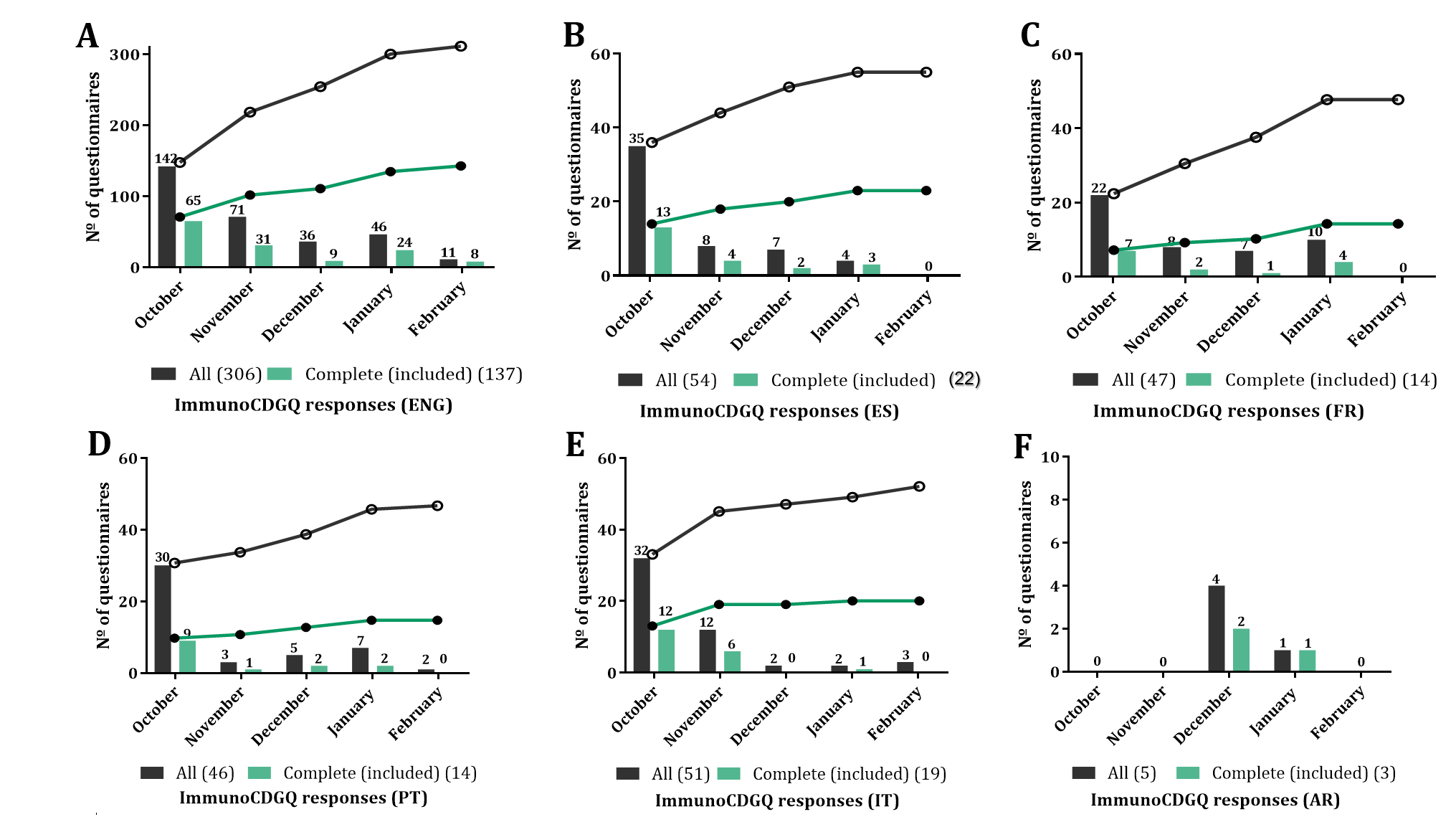


Supplementary figure 4. ImmunoCDGQ response tracking over time per language version. Initiated (black) and included (green) questionnaires of the A) English (ENG); B) Spanish (ES); C) French (FR); D) Portuguese (PT); E) Italian (IT) and F) Arabic (AR) versions.
